# Occurrence of *Campylobacter* spp. in Swedish calves, common sequence types and antibiotic resistance patterns

**DOI:** 10.1111/jam.14914

**Published:** 2020-11-12

**Authors:** I. Hansson, L.‐M. Tamminen, S. Frosth, L.‐L. Fernström, U. Emanuelson, S. Boqvist

**Affiliations:** ^1^ Department of Biomedical Sciences and Veterinary Public Health Swedish University of Agricultural Sciences Uppsala Sweden; ^2^ Department of Clinical Sciences Swedish University of Agricultural Sciences Uppsala Sweden

**Keywords:** antimicrobial resistance, *Campylobacter jejuni*, *Campylobacter hyointestinalis*, cattle, cgMLST, whole‐genome sequencing

## Abstract

**Aims:**

Cattle are the second most important cause of human campylobacteriosis, after poultry, but there are knowledge gaps regarding *Campylobacter* in cattle. This study examined the occurrence of *Campylobacter*, the species present, sequence types and antibiotic resistance in Swedish cattle.

**Methods and Results:**

Faeces samples collected from 154 calves on seven Swedish farms, and 69 follow‐up samples from a second collection occasion, were analysed. *Campylobacter* were isolated from 77% of calves at the first sampling, with *Campylobacter jejuni* as the most frequently isolated species. Animals kept on deep straw bedding were less likely to be colonized with *Campylobacter*. Whole‐genome sequencing of 90 *C. jejuni* samples resulted in 11 sequence types, among which ST‐19 and ST‐21 were most frequent. Antimicrobial resistance analyses showed that 46% of 142 isolates analysed were resistant to quinolones, while all isolates belonging to ST‐19, ST‐22 and ST‐441 were resistant to ciprofloxacin and nalidixic acid.

**Conclusions:**

*Campylobacter jejuni* was the species most frequently isolated in calves and a strong association was found between sequence type and antimicrobial resistance pattern.

**Significance and Impact of the Study:**

The high proportion of calves with quinolone‐resistant *Campylobacter jejuni* should be considered in a One Health perspective.

## Introduction

Campylobacteriosis is the most frequently reported zoonosis in many countries. There were almost 250 000 confirmed cases in Europe in 2018, representing more than 50% of all human cases of zoonotic infections reported in Europe (EFSA and ECDC 2019). However, these figures are likely to be underestimates and the true incidence is probably higher (Boqvist *et al*. 2018). Most cases of campylobacteriosis are sporadic, but outbreaks can occur. Poultry is an important reservoir of *Campylobacter jejuni*, and consumption and handling of broilers or broiler meat pose high risks of human campylobacteriosis (Rosner *et al*. 2017; Berthenet *et al*. 2019; Cody *et al*. 2019; EFSA and ECDC 2019). However, there are other sources of human campylobacteriosis (Sheppard *et al*. 2009; Mughini Gras *et al*. 2012; EFSA and ECDC 2019). For example, 54% of strong‐evidence outbreaks in the EU in 2017 were reported to be caused by milk, while in the US the most commonly identified sources of campylobacteriosis outbreaks 2010–2017 were milk‐associated (EFSA and ECDC 2018; CDC 2020). Outbreaks of campylobacteriosis have also been reported among cattle farm workers and visitors (Gilpin *et al*. 2008; Heuvelink *et al*. 2009; Lahti *et al*. 2017b). Cattle were identified as a *Campylobacter* reservoir for 21–55% of human cases in the Netherlands and France (Mughini Gras *et al*. 2012; Thépault *et al*. 2018b), and as the second most important cause of human campylobacteriosis in Denmark (Boysen *et al*. 2014).

Cattle are asymptomatic carriers of thermotolerant *Campylobacter* and may shed the bacteria intermittently in the faeces (Hakkinen and Hänninen 2009; Ramonaitė *et al*. 2013; Tang *et al*. 2017). This means that *Campylobacter* spp. can easily contaminate the udder and milk (Bianchini *et al*. 2014; Arthursson *et al*.^2018^; Hansson *et al*. 2020). However, there are still knowledge gaps regarding the epidemiology of *Campylobacter* spp. in cattle, for example, only eight EU member states reported monitoring data for *Campylobacter* in cattle in 2018 (EFSA and ECDC 2019). To our knowledge, there is no coordinated monitoring of *Campylobacter* spp. in cattle in any country.

Multi‐locus sequence typing (MLST) has become the standard for genetic analyses, e.g., to study transmission and risk factors (Lahti *et al*. 2017a, 2017b). There is a strong host‐genotype association of multi‐locus clonal complex (CC), sequence type (ST), and allele level, particularly within *C. jejuni*, which has a greater host signal than geographical signal (Sheppard *et al*. 2010). Most MLST analyses of *C. jejuni* in Sweden have been performed on isolates from chickens and humans. There is thus insufficient knowledge of common sequence types in isolates from cattle, and of the importance of cattle for campylobacteriosis in humans (Hansson *et al*. 2018).

There are also challenges with antimicrobial resistance (AMR) in *Campylobacter* spp., as reflected, for example, in increased resistance to quinolones such as ciprofloxacin and nalidixic acid (Riley *et al*. 2015; EFSA and ECDC 2017; Tang *et al*. 2017; CDC 2020). This problem has been highlighted by the WHO (2020). Ciprofloxacin resistance tended to increase over time among international travellers tested between 2007 and 2014 (Post *et al*.^2017^). In Sweden, resistance to ciprofloxacin in *Campylobacter* isolated from humans increased from 14% in 2014 to 61% in 2019 (Swedres‐Svarm 2019). In Swedish broilers, annual resistance of *C. jejuni* to fluoroquinolone varied between 4 and 24% during 2010–2018, although records of antibiotic sales show that fluoroquinolones were not used in commercial chicken production during this period (Swedres‐Svarm 2019). The low fluoroquinolone use is likely due to the ban on administering the antibiotic via feed or water, which makes distribution to poultry difficult. However, individual animals can still be treated under certain circumstances, for example, in dairy cattle there were 0·14 fluoroquinolone treatments per 100 completed/interrupted lactations in 2018 (Swedres‐Svarm 2019). However, there is limited information on AMR in *Campylobacter* strains isolated from cattle in Sweden.

To fill some of the knowledge gaps highlighted above, it is imperative to determine the occurrence and AMR patterns of *Campylobacter* spp. isolated from cattle, since this species can play an important role in the epidemiology of human campylobacteriosis. The aim of this study was thus to provide bacteriological and epidemiological knowledge on the occurrence, species, and sequence types of *Campylobacter* spp. in cattle, and to increase understanding of the AMR pattern of *Campylobacter* spp. isolates from Swedish cattle.

## Materials and Methods

### Study design and study population

**Figure 1:**
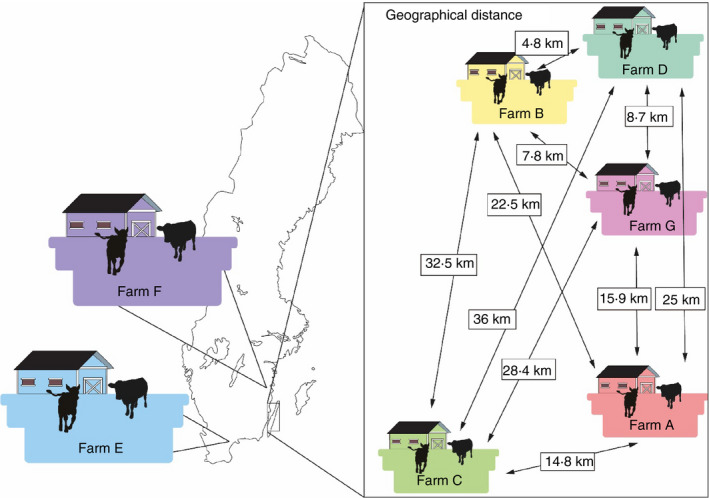
Location of farms included in the study and geographical distance between farms on the island of Öland (A, B, C, D, and G). The same color codes for the farms are used in Fig. 4.

This study was part of a larger study analysing Shiga toxin‐producing *Escherichia coli* (STEC) in Swedish calves, which was performed on seven dairy farms where presence of STEC had been confirmed through environmental sampling. Details of farm selection can be found in Tamminen *et al*. (2020). Five farms (A, B, C, D, G) were located on the island of Öland, Farm E in southern Sweden (Skåne) and Farm F in the south‐eastern county of Småland (Fig. 1). Farm size (total number of cattle) varied between 130 and 600 animals (Table 1). Faeces samples from 154 calves aged between 8 and 306 days (mean 113 days) were collected between April and November 2016. A detailed description of selection of animals for individual sampling can be found in Tamminen *et al*.(2020). In short, up to 26 animals per farm were selected by systematic random sampling in pens where STEC had been detected. If the pens on the farm contained fewer than 20 animals, all were sampled.

**Table 1:** Description of farms A–G, with herd size, pens sampled and age of calves sampled

Farm	Herd size (individuals)	Pen	Animals sampled	Animals in pen	Pen size (m^2^)	Age, (days) mean (SD)	*Campylobacter* positive	
							*n*	(%)
A	380	1	3	4	6·4	33 (2·5)	3	100
		2	3	4	6·3	34 (6·1)	3	100
		3	3	4	6·3	50 (7·1)	3	100
		4	3	4	6·3	51 (5·9)	3	100
		5	14	20	48	99 (29)	11	79
B	350	1	4	5	17	33 (4·4)	2	50
		2	7	7	13	78 (20)	4	57
		3	7	7	13	121 (21)	5	71
		4	1	1	13	42 (not applicable)	1	100
C	350	1	8	18	150	219 (13)	3	38
		2	4	8	26	177 (9·2)	4	100
		3	8	12	36	118 (42)	8	100
		4	1*	7	21	83 (not applicable)	0	0
D	600	2	7	8	22	101 (12)	7	100
		3	6	6	20	122 (3·5)	1	17
		4	7	7	22	141 (38)	2	29
E	300	1	3	4	7·2	61 (2·9)	3	100
		2	5	7	7·2	72 (9·7)	5	100
		3	5	7	7·2	92 (10)	5	100
		4	3	7	11	113 (46)	1	33
		5	7	6	10	232 (45)	6	86
F	135	1	12	12	25	66 (24)	9	75
		2	6	6	30	134 (44)	6	100
		3	7	13	128	195 (46)	4	57
G	130	1	3	3	5	135 (3·2)	3	100
		2	4	4	3·8	71 (7·8)	4	100
		3	2	3	5·5	143 (4·2)	2	100
		4	6	6	7·8	171 (25)	6	100
		5	3	3	2·7	43 (26)	3	100
		6	2	2	1·5	12 (4·9)	2	100
Total			154				119	77%

* A limited quantity of faeces was obtained from three calves, so analyses of STEC were given higher priority and the remaining faeces were used for analyses of *Campylobacter*.

**Figure 2:**
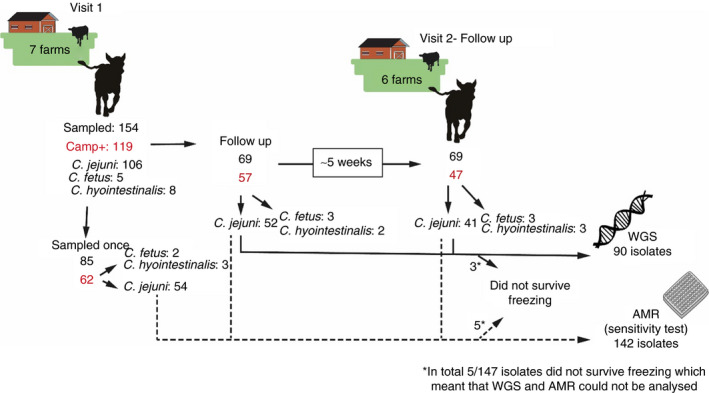
Overview of sampling of the seven herds, including first visits and follow‐up visits to six herds, and analyses performed on the isolates.

On six farms, a second sampling was performed 4–5 weeks after the first sampling. On this occasion, animals from which STEC had been isolated at the first sampling were included, together with 2–3 previously negative controls. Farm E was only visited once, since no animals tested positive for STEC in the first sampling (Fig. 2). Sampling of animals was performed in accordance with ethical approval granted by the regional ethics committee (Uppsala Djurförsöksetiska Nämnd, Dnr: C 85/15).

### Collection of faecal samples

Faecal samples from each animal were obtained from the rectum and transferred to 100 ml plastic jars. A new and clean pair of gloves were used for each sample and the jars were filled to a maximum of two‐thirds, in order to decrease the risk of the lid opening during transport. The samples were transported chilled to the National Veterinary Institute (SVA, Uppsala, Sweden). All packages reached the laboratory within 48 h. The samples were analysed by direct culture according to ISO 10272: Part 1C (2017). In brief, faecal contents were spread on modified charcoal‐cefoperazone‐deoxycholate agar (mCCDA) (Oxoid, Basingstoke, UK) and the plates were incubated at 37·0°C for 44 ± 4 h in microaerobic atmosphere generated by the use of CampyGen^TM^ (Oxoid). Identification of *Campylobacter* spp. was based on typical morphological aspects, white to grey colonies with metallic sheen and phase‐contrast microscopic observation with corkscrew movement according to ISO 10272: part 1 (2017).

### Species identification

Suspected *Campylobacter* colonies were re‐cultured on horse blood agar (SVA, Uppsala, Sweden) and incubated in microaerobic atmosphere at 37 ± 1°C for 44 ± 4 h. If suspected colonies had different macro‐morphological appearance, 2–3 isolates were re‐cultured for identification. Genus and species identification were performed from purified colonies on blood agar by matrix‐assisted laser desorption/ionization time of flight mass spectrometry (MALDI‐TOF MS), using a Microflex LT MALDI‐TOF mass spectrometer (Bruker Daltonics, Billerica, MA). Identification of *Campylobacter fetus* to subspecies were performed by sequencing, due to difficulties to distinguish between *Campylobacter fetus* subsp. *fetus* and *Campylobacter fetus* subsp. *veneralis* by MALDI‐TOF MS. At least one colony from each positive sample was stored in glycerol broth (15% glycerol and 85% serum broth) at −70°C.

### Antimicrobial susceptibility testing

Susceptibility to selected antimicrobial substances was assessed with VetMIC™ panel analysis systems: Camp EU, version 2013‐10 (SVA, Sweden), determining the antimicrobial minimum inhibitory concentration (MIC) against ciprofloxacin, erythromycin, gentamycin, nalidixic acid, streptomycin and tetracycline. Multi‐drug resistance was defined as resistance to three or more antibiotic classes. For example, resistance to ciprofloxacin and nalidixic acid was considered resistance to one antibiotic class (quinolones). Susceptibility testing was performed on 142 strains of *C. jejuni*. Not all strains isolated could be tested, because not all survived at −70°C. Reference strains of *C. jejuni* (CCUG 33560) were used as controls. Epidemiological cut‐off (ECOFF) values for determining susceptibility were obtained from the European Committee on Antimicrobial Susceptibility Testing (EUCAST) website https://www.eucast.org/mic_distributions_and_ecoffs/. The ECOFF values classify isolates with acquired reduced susceptibility as ‘non‐wild type’. In this paper, non‐wild type isolates are called ‘resistant’, in agreement with the Swedish Veterinary Antibiotic Resistance Monitoring report (Swedres‐Svarm 2018). This classification is relevant for monitoring purposes, but it should be understood that resistance defined in this manner does not always refer to clinical resistance.

### Whole‐genome sequencing

Whole‐genome sequencing (WGS) was performed on 90 *C. jejuni* isolates with at least seven isolates from each farm, including calves found to be colonized with *C. jejuni* on the two different sampling occasions. In order to increase the chances of getting different types of sequences from each farm, strains with at least one titre level difference of at least one antibiotic were chosen. All *C. jejuni* isolates were subcultured twice from single colonies on horse blood agar plates (SVA B341180; National Veterinary Institute) for 48 h at 41·5°C in microaerobic atmosphere, prior to DNA extraction. DNA was extracted using the EZ1 DNA Tissue Kit and the bacterial protocol for an EZ1 Advanced XL instrument (Qiagen, Hilden, Germany) according to the manufacturer’s instructions. The DNA was eluted in 100 *µ*l elution buffer from the kit and quantified using the Qubit ds DNA High Sensitivity Assay Kit on a Qubit® 2.0 Fluorometer (Invitrogen, Carlsbad, CA). Sequencing libraries were prepared using the Nextera XT DNA Library Preparation Kit (Illumina, San Diego, CA). The libraries were then sequenced on an Illumina NextSeq 500 system with 2 × 150‐bp paired‐end reads, using the NextSeq 500/550 Mid Output kit V2.5. The sequence reads generated were analysed using the Ridom SeqSphere + v6.0.9 software (Ridom GmbH, Münster, Germany). The genomes were assembled *de novo* using SKESA (Souvorov *et al*. 2018), through a pipeline script in Ridom SeqSphere+, with an average mean genome coverage of 183×. The MLST profiles were assigned using the scheme available at https://pubmlst.org/campylobacter/ (Jolley *et al*. 2018) through a *C. jejuni/coli* MLST task template in Ridom SeqSphere+. Core genome MLST (cgMLST) analysis was performed using the *C. jejuni/coli* cgMLST task template v1.3 in Ridom SeqSphere+, which contains 637 loci. A minimum spanning tree (MST) based on these 637 loci was generated in Ridom SeqSphere + using default parameter settings. Missing alleles were ignored in the pairwise comparisons. The MST was used to investigate the relationship between the isolates, while the default value of maximum difference of 13 cgMLST targets in the software was used to indicate relationship. ResFinder 3.2 (Zankari *et al*. 2012) was used to detect acquired antimicrobial resistance genes and chromosomal point mutations associated with antimicrobial resistance.

### Epidemiological determinants

At the time of the first sampling, information about the age of the sampled animals was collected from farm records. In addition, pen size was measured using a laser telemeter and the number of animals in each pen was noted. Each pen was also categorized according to type (group pen with deep straw bedding, pen with straw/sawdust bedding, or other, for example, slatted floors or free‐stall with cubicles). Hygiene (faecal contamination and wetness) was scored between 1 and 3 (1 = limited faeces visible, dry bedding, 2 = fecal contamination of bedding material clearly visible and/or bedding wet in part of the pen, 3 = faecal contamination visible and/or bedding wet in the whole pen).

### Statistical analysis

Differences in resistance to nalidixic acid and ciprofloxacin between farms were analysed by Fisher’s exact test, performed using a statistical program on the internet website ‘Social Science Statistics’ (https://www.socscistatistics.com). A probability level of *P* < 0·05 was considered statistically significant. Statistical analysis of the association between determinants and the dependent variable (calf testing positive or negative for *Campylobacter* spp.) was performed in R Statistical Software (R Core Team 2018). Univariable analysis of age and pen‐level risk factors was performed using Fisher’s exact test (categorical variables) and the Kruskal–Wallis rank sum test (continuous variables), using the package ‘tableone’ (Yoshida and Bohn 2019). This was followed by multivariable analysis by generalized logistic regression using lme4 (Bates *et al*. 2015). Numerical variables were scaled and centred before inclusion, and pen ID was included as a random effect. Variance inflation was investigated using the package ‘car’ (Weisberg and Fox 2011), with variance inflation >2·5 considered to indicate variance inflation. The model was reduced using likelihood ratio test and confounding was evaluated by re‐introducing each variable to the final model. Possible nonlinear associations of numerical variables were investigated using a generalized additive model before model reduction (Wood and Scheipl 2017). The outcome from STEC O157:H7 sampling (calf positive or negative) was forced into the final model to investigate the risk of bias due to a selection process based on presence of STEC O157:H7 in the environment.

## Results

### Occurrence of Campylobacter spp.

**Figure 3:**
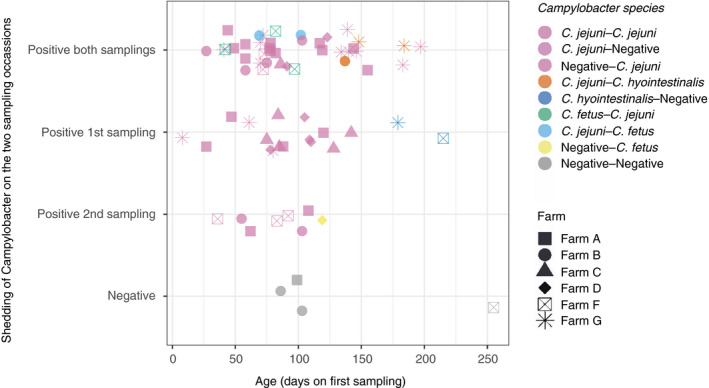
Results of bacteriological analyses for all 69 calves (from six farms) that were sampled twice, and*Campylobacter*spp. detected.

*Campylobacter* spp. were isolated from 119 (77%) of 154 calves at the first sampling (Table 2, Fig. 2). Sixty‐nine calves from six farms were sampled twice and *Campylobacter* spp. were isolated at both samplings from 39 (57%) calves. *Campylobacter jejuni* was the most frequently isolated species, isolated from 106 (67%) calves, followed by *Campylobacter hyointestinalis* isolated from eight (5%) calves and *C. fetus* subsp. *fetus* from five calves (3%). In 31 (45%) calves, *C. jejuni* was isolated on both sampling occasions (Fig. 3). Median age at the first sampling was 103 days (around 3·5 months).

**Table 2:** *Campylobacter* spp. isolated from faeces samples taken from 154 calves on seven different farms (A‐G) in Sweden

Farm	*Campylobacter jejuni*	*Campylobacter fetus* subsp. *fetus*	*Campylobacter hyointestinalis*	*Campylobacter* not detected
A	19 (73%)	1 (4%)	3 (12%)	3 (12%)
B	12 (63%)	0	0	7 (37%)
C	14 (67%)	0	1 (5%)	6 (18%)
D	10 (50%)	0	0	10 (50%)
E	20 (83%)	0	0	3 (7%)
F	13 (52%)	4 (16%)	2 (8%)	6 (24%)
G	18 (90%)	0	0	2 (10%)

### Associations between Campylobacter spp. and epidemiological determinants

The univariable analysis showed significant differences in age, number of animals in a pen, pen size, and pen type between calves testing positive for *Campylobacter* spp. and those testing negative (Table 3). Pen hygiene and number of animals in a pen introduced variance inflation in the multivariable model, most likely due to high correlation with pen type and pen size, and were excluded from the final model. After model reduction, pen type was the only variable left in the model. The results indicated that calves in pens with straw/sawdust bedding or concrete/rubber surfaces were more likely to test positive for *Campylobacter* spp. (odds ratio (OR) = 8 and 11 respectively; *P* = 0·018) than calves in pens with deep straw bedding. Re‐introduction of removed variables indicated that some of this effect may have been due to pen size and number of animals in a pen, since these variables influenced the estimates of pen type. On accounting for pen type, young age was not associated with presence of *Campylobacter* spp., but it should be noted that there may be systematic differences in age between pen type (e.g. calves kept on straw were younger than calves on deep straw bedding), which may have biased this result. The generalized additive model did not indicate nonlinear associations between presence of *Campylobacter* spp. and the determinants. Univariable and multivariable analysis showed no association between *Campylobacter* spp. and STEC O157:H7.

**Table 3:** Outcomes of *Campylobacter* testing of calves in pens according to determinants

	*Campylobacter*spp.		
	Negative	Positive	*P* ^†^
Number (*n*)	35	119	
Age in days (median [IQR*])	119 [89, 178]	97 [64, 141]	0·04
Positive for STEC O157:H7 (%)	6 (17)	23 (19)	1
Number of animals in pen (median [IQR])	7 [7, 13]	7 [4, 12]	0·03
Pen size in m^2^ (median [IQR])	22 [17, 48]	13 [7, 30]	0·003
Pen type (%)			<0·001
Deep straw	18 (51)	17 (14)	
Straw/sawdust	14 (40)	83 (70)	
Other (concrete/rubber/slatted)	3 (9)	19 (16)	
Pen hygiene (%)			0·1
Clean and dry pen	13 (37)	67 (56)	
Dirty and/or partly wet pen	4 (11)	10 (9)	
Dirty and wet pen	18 (52)	42 (35)	

* Interquartile range.

^†^ *P*‐values derived from univariable analysis using Fisher’s exact test (categorical variables) or the Kruskal–Wallis rank sum test (numerical variables).

### Antimicrobial susceptibility

Antimicrobial resistance to ciprofloxacin was detected in 66 (46%) of the 142 *C. jejuni* isolates that could be tested. No resistance to either gentamycin or erythromycin was recorded (Table 4), and none of the 142 isolates showed multi‐drug resistance.

**Table 4:** Distribution in terms of antimicrobial minimum inhibitory concentration (MIC) of *Campylobacter jejuni* in calves that tested positive for the species (*n* = 142)

Antibiotic	MIC (mg l^−1^)*										
	≤0·12	0·25	0·5	1	2	4	8	16	32	64	>64
*Quinolones*											
Ciprofloxacin	48	6		2			36	8			
Nalidixic acid					7	28	16	2		1	46
*Macrolides*											
Erythromycin				95	4	1					
*Aminoglycosides*											
Gentamycin		24	64	11	1						
Streptomycin			1	50	40	8		1			
*Tetracyclines*											
Tetracycline			83	7	1					9	

* White fields denote range of dilutions tested for each antibiotic and vertical bold lines indicate cut‐off values used to define resistance. MICs equal to or lower than the lowest concentration tested are given as the lowest tested concentration.

There was variation between the farms regarding AMR of the *C. jejuni* strains detected. The MIC distributions for nalidixic acid and ciprofloxacin differed significantly between herds (*P* < 0·001) (Table 4). All 20 strains isolated from the calves on farm E were resistant to ciprofloxacin and nalidixic acid. In contrast, only one of the 19 strains of *C. jejuni* isolated from the calves on farm F showed resistance to ciprofloxacin and nalidixic acid, while the remaining 18 strains were sensitive to all antimicrobials tested (Table 5). There was no association between quinolone resistance (resistance to ciprofloxacin and nalidixic acid) and pen type or animal age.

**Table 5:** Sensitivity and resistance to antibiotics of 142 strains of *Campylobacter jejuni* isolated from calves on seven farms (A–G) in Sweden

Farm	No. of *C. jejuni*	Sensitive to all tested antibiotics (%)	Number of resistant isolates			
			Nal	Nal + Cip	Nal + Cip + Str	Nal + Cip + Tet
A	20	5 (25%)	1	10	0	4
B	28	24 (79%)	0	4	0	0
C	14	5 (36%)	0	1	0	8
D	13	7 (54%)	0	4	1	1
E	20	0 (0%)	0	20	0	0
F	19	18 (94%)	0	1	0	0
G	28	16 (57%)	0	12	0	0

Nal = nalidixic acid, Cip = ciprofloxacin, Str = streptomycin, Tet = tetracycline.

### Whole‐genome sequencing

**Figure 4:**
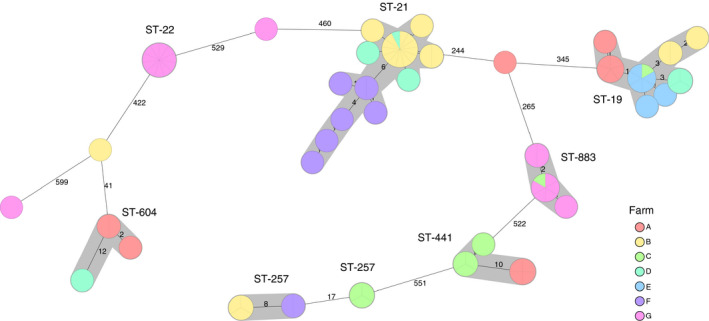
Minimum spanning tree (MST) generated for 90*Campylobacter jejuni*isolates from seven cattle farms (A–G) in Sweden, based on core genome multi‐locus sequence typing (cgMLST) data. MST calculated by pairwise comparison of 637 loci, with missing values ignored. Nodes corresponding to sequenced isolates are colored according to cattle farm. Grey background indicates genetically related isolates (maximum difference of 13 cgMLST targets). Sequence type (ST) is given if at least two isolates share the same ST. Values on the lines between nodes represent allelic differences. Line length is not proportional to the numbers.

The two most frequently isolated sequence types were ST‐21 (29%) and ST‐19 (22%), both belonging to clonal complex CC‐21 (Table 6). For one of the farms (E), all seven sequenced isolates belonged to ST‐19, whereas 2–4 STs were identified on the other six farms (Fig. 4).

**Table 6:** Clonal complex (CC) and multi‐locus sequence type (MLST) of 90 *Campylobacter jejuni* isolates from faeces samples taken from calves on seven different farms (A–G) in Sweden

CC	MLST	Farm							Total
		A	B	C	D	E	F	G	
21	19	6	3	1	3	7			20
21	21		16		3		7		26
22	22							11	11
257	257		3	3			2		8
NA	441	4		4					8
42	604	3			1				4
21	883			1				8	9
61	3936							1	1
NA	6591							1	1
21	9830	1							1
42	10227		1						1

NA, not assigned.

Among the 69 calves that were sampled twice, *C. jejuni* were isolated from 31 calves on both occasions. Fourteen of these *C. jejuni* had the same sequence type and resistance pattern on both sampling occasions, five of the calves had *C. jejuni* with the same sequence type but different resistance pattern, and the isolated *C. jejuni* were of different sequence types in 12 of the calves.

A distinct relationship was observed between sequence type and AMR pattern, since the eight different sequence types, with one exception, showed exactly the same pattern (Fig. 4). Of the 20 strains belonging to ST‐19, 18 were resistant to nalidixic acid and ciprofloxacin, one to nalidixic acid, ciprofloxacin and streptomycin, and one to nalidixic acid, ciprofloxacin and tetracycline. All eight strains of ST‐441 were resistant to nalidixic acid, ciprofloxacin and tetracycline, and all 11 strains of ST‐22 were resistant to ciprofloxacin and nalidixic acid. All strains belonging to ST‐21, ST‐257, ST‐604, ST‐883, ST‐3936, ST‐6591 and ST‐10227 were sensitive to all six antimicrobial substances tested.

## Discussion

This study confirmed that *Campylobacter* spp. are often part of the intestinal microbiota in cattle. The high level of detection, 77% in faeces samples, is in line with findings that 53–83% of cattle tested are colonized with *Campylobacter* spp. (Ramonaitė *et al*. 2013; Tang *et al*. 2017; Jaakkonen *et al*. 2019; Waldner *et al*. 2019; Hansson *et al*. 2020). The experimental set‐up was designed to analyse occurrence and shedding of STEC, which might potentially introduce sampling bias in analysis of *Campylobacter* spp. However, there was no association between STEC status and presence of *Campylobacter* spp., and the results on occurrence of *Campylobacter* spp. correspond to those presented in other studies. In this study, *C. jejuni* was identified in 67% of the samples, *C. hyointestinalis* in 5%, and *C. fetus* subsp. *fetus* in 4%. These proportions are in agreement with results presented in other studies, in which *C. jejuni* was the dominant species (20–68%), followed by *C. coli* (0–24%), *C. hyointestinalis* (0–11%), *C. lari* (0–1%), and *C. fetus* subsp. *fetus* (0–1%) (Hakkinen *et al*. 2007; Ramonaitė *et al*. 2013; Thépault *et al*. 2018a; Hansson *et al*. 2020). Calves kept on deep straw bedding were less likely to be colonized with *Campylobacter* spp., despite these pens often being associated with poor hygiene. However, pen type was also associated with calf age, pen size and number of animals in the pen, and the effects of these variables cannot be separated due to confounding. Thus, more studies on how environmental and management factors influence presence and survival of *Campylobacter* spp. are needed to reveal the underlying causal relationship, and to estimate the potential for using management‐ and environment‐related measures to reduce presence of the pathogen.

In this study, only one colony from each sample was subcultured for species identification. However, in faeces samples from three calves, more than one *Campylobacter* species was isolated. It is likely that more calves were colonized with at least two different *Campylobacter* species or sequence types, for example, a French study isolated at least two *Campylobacter* species from 10% of the cattle tested (Thépault *et al*. 2018a). Different methods of cultivation have also been shown to favour different species of *Campylobacter* spp., and thereby influence isolation (Ramonaitė *et al*. 2013; Hansson *et al*. 2020). If more than one colony had been selected and if additional culturing steps had been included, more species and sequence types per calf would probably have been obtained in the present study.

MLST resulted in 90 *C. jejuni* isolates belonging to 11 different STs, grouped within five CC, two of the ST´s have however not been assigned to a CC yet. The most common clonal complex was CC‐21, followed by CC‐22 and CC‐257. ST‐21 was found to dominate, as reported previously in other studies on cattle (de Haan *et al*. 2010; Bianchini *et al*. 2014; Thépault *et al*. 2018b; Aksomaitiene *et al*. 2019). Some genotypes have also been more frequently isolated from humans, for example, ST‐21 was the type most frequently isolated from humans with campylobacteriosis during 2011–2012 in Sweden, while the highest proportion of hospitalized cases resulted from infection with ST‐257 (Harvala *et al*. 2016). In a study by Jaakkonen *et al*.(2019), some *C. jejuni* strains from cattle, such as ST‐883 and ST‐1080, persisted for at least 11 months, whereas other *C. jejuni* types were found sporadically. The longest time interval between repeated sampling occasions in the present study was 34 days, and the same STs were found on both occasions in 14 calves.

There was high similarity in terms of both ST and AMR between the farms. This was partly expected, considering that five of the farms included in the study were situated on the island of Öland and were only 4·8–36 km apart (Fig. 1). Between‐farm contacts (such as transporting animals together, animal contact on pasture, sharing manure spreader, and farmers visiting each other) are also common on the island (Tamminen *et al*. 2019). Additionally, Öland hosts many migratory wild birds that could be colonized with *Campylobacter* and contribute to bacterial spread to other individuals (Broman *et al*. 2004; Söderlund *et al*. 2019). Migratory wild birds can also transfer antimicrobial resistance through horizontal gene transfer (Sjölund *et al*.^2008^). Öland is also a popular recreation area with a large number of visitors that may contribute to indirect spread of *Campylobacter* by transferring cattle or bird faecal material on, for example, boots and vehicles. However, the strains isolated from the two farms located in other regions of Sweden (E, F) were closely related to the strains isolated from the Öland farms, indicating that geographical distance is not associated with genetic distance of *Campylobacter*. Thus, transmission routes other than between‐farm spread may be important to consider in future studies.

The occurrence of ciprofloxacin and nalidixic acid resistance in this study was remarkably high (46% and 47%). The high level of resistance to quinolones is difficult to interpret, but could be due to the hyper‐mutable nature of *Campylobacter*. Point mutations in the quinolone resistance‐determining region of *gyrA* gene are most often responsible for resistance to fluoroquinolones (Payot *et al*.^2006^). The strong association that was found between specific genotypes and resistance to antimicrobials have also been observed in studies of chicken isolates of *C. jejuni* (Habib *et al* 2009; Wirz *et al*.^2010^). Previous studies in Sweden of *C. jejuni* isolates from faeces from healthy cattle, sampled at slaughter during four different years between 2001 and 2015, showed annual resistance of 2–21% to ciprofloxacin and 2–23% to nalidixic acid (Swedres‐Svarm, 2018). The difference between the studies is difficult to explain, but could be due to different sample sizes and sampling frames. For instance, the cattle in the present study were younger and only from dairy farms, whereas those studied by Swedres‐Svarm (2018) included older animals and cattle from both dairy and beef farms. The use of quinolones in livestock production is restricted under Swedish Board of Agriculture regulations on medicine and drug use (SJVFS 2013:42). The main clinical indication for treatment of cattle with quinolones is mastitis (SVS 2015), and obviously dairy cows are more likely to suffer from mastitis than beef cows. This could be another explanation for the different results obtained in this and previous studies. Additionally, different age groups are present more often in dairy herds compared with beef herds, and transmission of resistant bacteria to younger animals through the environment could be facilitated, unless an all in‐all out system is used. None of the isolates was resistant to macrolides (erythromycin), the drug of choice for treatment of human campylobacteriosis. Low resistance to macrolides has also been found in the Swedish human population, with <1% of *C. jejuni* isolates from humans being resistant to erythromycin (Swedres‐Svarm, 2019).

Antibiotic resistance, particularly to quinolones and macrolides, in thermotolerant *Campylobacter* spp. is considered a serious threat to public health, as clinical treatment of campylobacteriosis may require use of those antibiotics. A high proportion of cattle colonized with quinolone‐resistant *C. jejuni* could result in continuous contamination of the environment and food products, which should be considered in a One Health perspective.

## Conflict of Interest

None of the authors declares a conflict of interest.

## Author contributions

I.H. and S.B. contributed to conceptualization. I.H., L‐M.T, S.F. and S.B. contributed to data curation I.H., L‐M.T., S.F. and L‐L.F. contributed to formal analysis and investigation. I.H. U.E, L‐M.T. and S.B. contributed to funding acquisition. I.H. L‐M.T. S.F. L.‐L.F. U.E. and S.B. contributed to methodology. L‐M.T. and S.F. contributed to visualization. I.H. contributed to writing—original draft. I.H., L‐M.T., S.F., U.E. and I.H. contributed to writing—review & editing. All authors have read and agreed to the published version of the manuscript.
